# Evaluation of Molecular Simulations and Deep Learning Prediction of Antibodies’ Recognition of TRBC1 and TRBC2

**DOI:** 10.3390/antib12030058

**Published:** 2023-09-17

**Authors:** Xincheng Zeng, Tianqun Wang, Yue Kang, Ganggang Bai, Buyong Ma

**Affiliations:** 1 Engineering Research Center of Cell & Therapeutic Antibody (MOE), School of Pharmacy, Shanghai Jiao Tong University, Shanghai 200240, China; zeng_xincheng@163.com (X.Z.); bggyxyky@163.com (G.B.); 2 Shanghai Digiwiser Biological Inc., Shanghai 200240, China; mia01.wang@fapon.com (T.W.); yue.kang@fapon.com (Y.K.)

**Keywords:** antibody, artificial intelligence, antibody design, free energy perturbation, molecular dynamics simulations

## Abstract

T cell receptor β-chain constant (TRBC) is a promising class of cancer targets consisting of two highly homologous proteins, TRBC1 and TRBC2. Developing targeted antibody therapeutics against TRBC1 or TRBC2 is expected to eradicate the malignant T cells and preserve half of the normal T cells. Recently, several antibody engineering strategies have been used to modulate the TRBC1 and TRBC2 specificity of antibodies. Here, we used molecular simulation and artificial intelligence methods to quantify the affinity difference in antibodies with various mutations for TRBC1 and TRBC2. The affinity of the existing mutants was verified by FEP calculations aided by the AI. We also performed long-time molecular dynamics simulations to reveal the dynamical antigen recognition mechanisms of the TRBC antibodies.

## 1. Introduction

The TCRα/β recognizes foreign (peptide) antigens peptide-loaded MHC (pMHC) and then activates many signal transduction cascade reactions, regulating cell survival, proliferation, differentiation, and cytokines generation. TCR activation is fundamental to the immune response and plays essential roles not only in the protection against invading microorganisms but also in the suppression of cancer. Like antibodies, TCR diversity is generated by somatic recombination of variable (V), diversity (D), joining (J), and constant (C) regions. There are two β-chain constant region genes, TRBC1 and TRBC2. While each TCRα/β carries either TRBC1 or TRBC2 in a mutually exclusive manner, the normal T cell population comprises a mixture of individual cells, either expressing TRBC1 or TRBC2. However, the cancer T-cell population exclusively expresses either TRBC1 or TRBC2. Using antibodies to specifically target TRBC1 (TCRαβ1) or TRBC2 (TCRαβ2) (in the case of a TRBC1+ T cell malignancy or the case of a TRBC2+ malignancy, respectively) has been proposed to be a strategy to eradicate all cells of malignant cancer [1].

**Figure 1:**
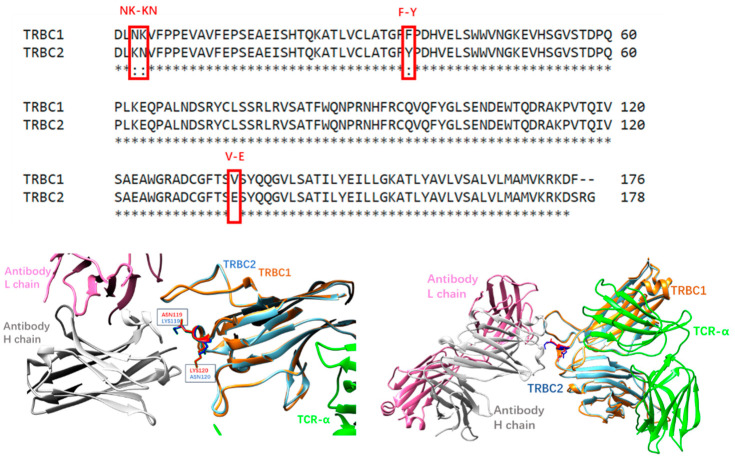
(**Upper panel**): sequence alignment of TRBC1 and TRBC2 constant regions. (**Lower left panel**): JOVI-1 antibodies recognize the N3K4/K3N4 region. (**Lower right panel**): crystal structures of JOVI-1-TRBC1/2 complexes have similar conformations.

TRBC1 and TRBC2 differ in four residues located in three different structural regions (Figure 1): NK or KN in positions 3-4; F or Y in position 35; and V or E in 134. Since F35 is buried and V134 is in the transmembrane domain, the only possible epitope to differentiate TCRαβ1 or TCRαβ2 is the N3K4/K3N4. The trivial change in epitope tests the sensitivity and specificity of antibodies to bind TCRαβ1 or TCRαβ2 selectively. Using a transgenic mouse line expressing a human V beta 3 C beta 1 TcR, monoclonal antibody JOVI-1 was obtained [2] and found to recognize only TRBC1-TCR (TCRαβ1) and not TRBC2-TCR (TCRαβ2) [2,3]. The JOVI-1 can be used as anti-TRBC1 antibody-based flow cytometric detection of T-Cell clonality [4,5,6] and, more importantly, to construct anti-TRBC1 CAR-T cells that kill TRBC1 expressing normal and malignant T cells while sparing TRBC2 restricted cells [3]. Other CAR-T targeting TRBC1 [7] or similar TRBV family members [8] expressed in malignant T cells were also reported. Interestingly, based on computational structure-guided specificity redirection of JOVI-1, three mutations of the JOVI-1 VH chain (T28K, Y32F, A96N) successfully switched JOVI-1 to bind the highly homologous target TRBC2-TCR (TCRαβ2) [9], opening another opportunity for the T-cell malignancy treatments.

Computational antibody designs have been developed to help antibody designs [10,11,12,13,14,15,16], including molecular mechanical energy-based de novo design [11], molecular dynamics simulations [14,17,18,19], structure-based antibody prediction and similarity measure of antibody–antigen interfaces [20,21]. Molecular docking and subsequent MD simulation have also been used to predict the binding mode of JOVI-1 with TRBC1 [19]. Recently, various deep learning-based machine learning approaches have pushed the advances of the antibody engineering [22,23,24,25,26].

In this study, we used sequence-based antibody affinity prediction, molecular dynamics simulation, and free energy perturbation to analyze the family of antibody JOVI-1 and their selective bindings to TCRαβ1 and TCRαβ2. While training on vast variations in antibody–antigen sequence and mutations, our machine learning model can predict the trend of change in binding affinity; however, it does not have the precision to distinguish epitope switch of N3K4/K3N4 in TRBC1 and TRBC2, respectively. Molecular dynamics simulations of fourteen variations in JOVI-1 mutant TRBC1/2 complexes indicated that the antibody–antigen complex with a binding affinity of less than 8 kcal/mol tends to disassociate in simulation. Finally, our free energy perturbations of amino acid mutation reproduced the selective binding trend of JOVI-1 family antibodies.

## 2. Materials and Methods

### 2.1. AI Model Training and Prediction of Antibody–Antigen Binding Affinity

We designed a listwise ranking model specifically for predicting changes in affinity based on mutations. This model is trained on SKEMPI [27] Antibody–Bind (AB-Bind) [28] datasets, both curated antibody–antigen complexes with single-site and multi-site mutations and corresponding free energy change values (∆∆G). The training dataset comprises a total of 1413 antibody–antigen pairs, each annotated by binding free energy values (affinity). Our approach involves training the transformer encoder layers using antibody and antigen sequences as inputs. The pre-processed training data comprise wild-type antibody–antigen pairs and their corresponding mutant, organized into separate lists. Each list consists of antibody variants binding to the same antigen. The binding affinity changes are normalized to (0,1) as the “relevance score”, akin to the idea of “relevance” in searching problems.

Our model, therefore, focuses on fitting the relevance scores rather than actual ∆∆G values with the use of normalized discounted cumulative gain (NDCG) loss function. Pre-trained language models AntiBERTy [29] and ProtBert [30] are utilized to generate embeddings for antibody and antigen sequences. The ranking model is expected to capture the enabling features that contribute to binding affinity changes at the amino acid level.

The correlation between experimental measurements and predicted ranking scores was evaluated with the Pearson correlation coefficient (for linear correlation) and the Spearman coefficient (for ranking correlation) in Table 1. The proposed Digiwiser model yields Pearson correlations ranging from 0.71 to 0.89 on five-fold validations, outperforming most conventional (structure-based) in silico approaches [28].

### 2.2. Molecular Dynamics Simulations and Analysis

The initial structures of simulations were taken from crystal structures listed in Table 2, with disulfide bonds constructed accordingly. For the chains of antibody and antigen, the N termini and C termini were charged (NH^3+^ and COO^−^ groups, respectively). The missing residues in crystal structures were added using the CHARMM-GUI input generator [31]. The systems were then solvated by TIP3 water molecules with a minimal distance of 15 Ǻ from any protein atom to any edge of the water box. Sodium and chloride ions were added to neutralize the system to a total concentration of ~150 mM. The resulting solvated systems were energy-minimized for 50,000 steepest descent steps:q. In the heating stage, each system was gradually heated to 50 K and then to 250 K. In the production stage, all simulations were performed using the NPT ensemble at 300 K, with a timestep of 2 fs. The particle mesh Ewald (PME) method was used to calculate the electrostatic interaction, and the van der Waals interactions were calculated using a cutoff of 12 Å. All MD simulations were performed using the amber20 software (University of California, San Francisco, CA, USA) [32] with CHARMM36 force field [33]. MD trajectories were saved by every 0.1 ns for analysis. A summary of all simulation systems is given in Table 2.

RMSD/RMSF calculation and Correlation analysis: The root mean squared deviation (RMSD) and root mean square fluctuation (RMSF) for the backbone of each structure were calculated by VMD. Correlations between all the residues were analyzed for the stable MD trajectory using the normalized covariance of the motion of protein residues [34], ranging from −1 to 1. If two residues moved in the same (opposite) direction in most of the frames, the motion was considered (anti-)correlated, and the correlation value was close to 1 or −1. If the correlation value between two residues was close to zero, they were generally uncorrelated. The correlation evaluation was performed using the program CARMA [35]

### 2.3. NAMD-Free Energy Perturbation Protocol

**Scheme 1:**
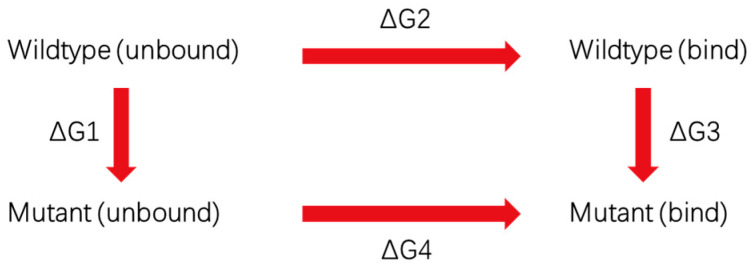
The thermodynamic cycle to calculate free energy change.

Free energy perturbation (FEP) calculations were performed starting from four different crystal binding poses (7amp, 7amq, 7amr, 7ams). In total, two kinds of systems were set up by using the CHARMM-GUI program. One was the antibody (light and heavy chain) in solvent (unbound state). The others were the TRBC and TCR-bound with antibody (bound state), both at 298 K and 1 atm. The system was solvated and ionized with the same condition described in the MD simulation section. The mutation of antibodies was conducted using the VMD program. NAMD all-atom molecular dynamics simulations [36] with CHARMM36 force field [33] were performed to equilibrate both kinds of systems using an NVT ensemble after a brief energy minimization. The dual-topology methodology and the soft-core potentials were applied to overcome endpoint singularities in the FEP simulation. The FEP method uses a thermodynamic coupling parameter λ, which is in the range of 0 to 1, to scale the potential energy change in mutating a residue on the antibody. In this study, one FEP calculation included 16 windows, ranging from λ values of zero to 1 (with a gap of 0.0625) for a total of 32 simulations per mutation since we ran both forward and backward simulations in each window to check the consistency of each simulation. The time step was 2.0 fs. During the FEP simulations, each window was equilibrated for 0.2 ns before collecting the FEP data based on the subsequent 0.8 ns simulations. After that, the change in free energy is calculated with Scheme 1 and Equation (1).

(1) $$\Delta \Delta G_{mut_{i}}=\Delta G_{4}-\Delta G_{2}=\Delta G_{3}-\Delta G_{1}$$

## 3. Results

### 3.1. AI Prediction of Mutation Effects on JOVI-1’s Recognition of TRBC1 and TRBC2

The training of our listwise ranking model for predicting affinity change (ΔΔG) was described in the Methods section. Using our trained prediction model, we tested the sequence-based antibody binding affinity change upon point mutations. In the prediction of TRBC1 binding ranking, we used the HuJovi-1 Fab TRBC1 complex and the HuJovi-1/KFN TRBC2 complex as references to probe TRBC1 and TRBC2 binding ranking, respectively. Table 3 lists experimental binding affinity changes and AI model-predicted mutation ranking scores. The lower number responds to mutation with higher binding affinity. As can be seen from Table 3, this model has a better Pearson correlation for TRBC1 binding than binding to TRBC2. However, as for Spearman ranking correlation, those antibody bindings to TRBC2 have a better ranking correlation between experimental binding affinity and AI model-predicted preference. In the TRBC1 group, this model produces a notable correlation (Pearson = 0.543), with a comparatively weaker correlation (Pearson = 0.272) for the TRBC2 group. The Spearman score for the TRBC2 group demonstrates robust ranking performance (Spearman = 0.899), whereas the TRBC1 group exhibits a relatively lower correlation (Spearman = 0.143). The overall correlation coefficient of the ranking score for all entries in Table 2 has an R^2^ value of 0.1. We explored the potential explanations for this observation in the Discussion and Conclusions. This ΔΔG ranking prediction model was trained from a dataset with different antibody–antigen pairs, but for comparable antibody mutations, the antigens have identical sequences. Therefore, it seems that this model has a better ability to rank antibody mutations with the same antigens. However, for the system with a slightly changed antigen sequence, these models have difficulty recognizing the small variation. This is the reason that for a given antibody, the rank scores for TRBC1 and TRBC2 are almost identical (Table 3). Thus, in future training of antibody–antigen AI models, it is important to include similar antigens as well.

### 3.2. Prediction of Mutation Effects on JOVI-1’s Recognition of TRBC1 and TRBC2 with Free Energy Perturbation

Free energy perturbation simulations of key mutations controlling the binding preference of TRBC1 and TRBC2 were performed based on the protocol detailed in the Methods section. The results are listed in Table 4. These three mutations represent four types of amino acid changes. T28K switches a neutral residue to a positively charged Lys; K28R has a small mutation effect since it switches between similar amino acids Lys and Arg. Y32F removes the hydroxyl group from Tyr, and A96N allows for the addition of hydrogen bonding with TRBC1/TRBC2. Due to charge repulsion between TCRβ1 and K120, the T28K mutation decreases binding affinity with TRBC1 by 0.56 kcal/mol but increases TRBC2 binding by 0.33 kcal/mol since the position now is N120 in TRBC2. Generally speaking, the free energy perturbation calculations involving charged residues usually have larger errors. In our FEP calculations, the error for the T28K mutation in TRBC1 binding is 0.1 kcal/mol but larger in TRBC2 binding (1.85 kcal/mol).

The Y32F mutation calculations have different simulation behaviors. The seemingly small change in Tyrosine-to-Phenylalanine mutation has normal accuracy in the single mutation system of Mut5a, which directly mutates Tyr32 to Phe from the 7AMP structure. The experimentally free energy change is 3.04 kcal/mol, and the FEP number is 2.24 kcal/mol, with an error of 0.80 kcal/mol. However, the combination of T28K and Y32F in mut2a/mut2b systems caused large errors in our FEP calculations (not listed). The T28/Y32 are the most important residues to maintain Jovi-1’s selective binding of TRBC1/TRBC2. T28K/Y32F mutations cause large-scale reorganization of the hydrogen bonding network between Jovi-1 and TRBC’s interfaces (see next section for details). Therefore, we changed our FEP protocol to run the reversed mutation of N96A from the KFN mutant, and that mutation was equivalent to T28K/Y32F mutations from the original Jovi-1 antibody. As a result, the FEP energy changes for mut2a/2b systems are satisfactory.

K28R mutation has very little effect on Jovi-1’s binding of TRBC1/TRBC2. Our FEP calculation fairly reproduced the experimental result of −0.32 kcal/mol in TRBC1 binding, with an error of only 0.13 kcal/mol. However, the error for TRBC2 binding of this mutation is larger (2.58 kcal/mol).

The FEP energies for A96N (A100N in our residue numbering) mutations in both Mut6a (direct A96N mutation from 7AMP) and mut6b (A96N mutation from 7AMQ) have normal accuracies, with errors of 0.72 kcal/mol and 2.01 kcal/mol, respectively.

A close look at Table 4 reveals that our FEP calculations for the TRBC1 system have smaller errors (average 1.28 kcal/mol) than the TRBC2 system (average error 2.09 kcal/mol). This could reflect that JOVI-1’s binding to TRBC2 is more sensitive to mutation than TRBC1 binding. Indeed, JOVI-1 was initially generated against the TRBC1 [2] and engineered to bind the TRBC2 [9].

### 3.3. Dynamic Features of JOVI-1 and Mutants’ Recognition of TRBC1 and TRBC2

We have systematically studied 18 complexes of JOVI-1 (and mutants) with TRBC1/TRBC2 using MD simulations up to 1000 ns, and we listed the simulation system and simulated antibody–antigen stabilities in Table 2. Interestingly, three complexes with the lowest binding affinities (JOVI-1 TRBC2, mut1b JOVI-1/T28 TRBC2, and mut3a JOVI-1/KFN TRBC1) disassociated at different simulation times (Table 2). Mut6b (JOVI-1/A96N TRBC2) also disassociated at around 600 ns. The stability of these complexes during our MD simulation is consistent with switching selectivities from TRBC1 to TRBC2 with the mutations that occurred.

**Figure 2:**
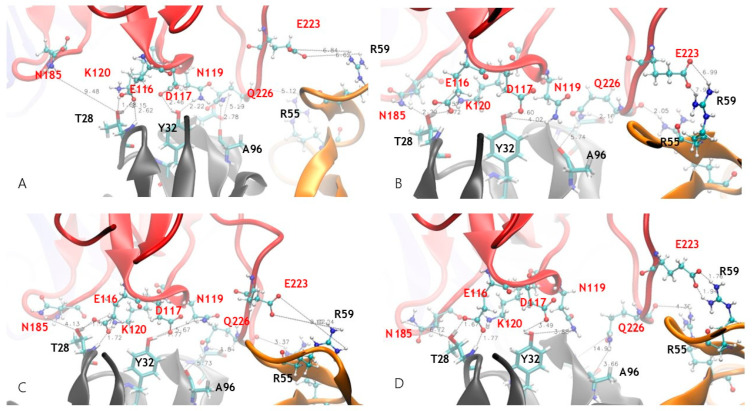
Dynamics hydrogen bond network formed between TRBC1 b-chain and JOVI-1 antibody at different simulation times. (**A**) Starting crystal structure. (**B**) Snapshot at 50 ns. (**C**) Snapshot at 100 ns. (**D**) Snapshot at 200 ns.

The crystal structures of JOVI-1 antibodies’ complexes with TRBC1/TRBC2 explain many structural features for JOVI-1’s selectivities [9]. However, there are several results that are not obvious from the crystal structures only. For example, one of JOVI-1’s preferences for TRBC1 over TRBC2 is that the heavy chain T28 of JOVI-1 forms a hydrogen bond with L120 in TRBC1 (Figure 2A), which is absent in TRBC2. However, the JOVI-1 T28K mutant (mut1a and mut1b), which cannot form hydrogen bonds with the L120, still maintains a high affinity with TRBC1 and has a decreased binding affinity with TRBC2, in comparison to the parent wt JOVI-1 (Table 2).

**Figure 3:**
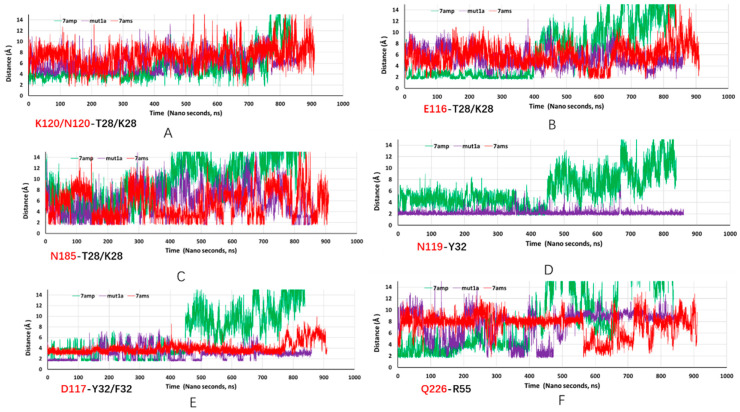
Trajectories of key hydrogen bonds from MD simulations of JOVI-1 TRBC1 (7amp, green line), JOVI-1/T28K TRBC1 (mut1a, purple line), and JOVI-1/KFN TRBC2 (7ams, red line). Residues in TRBC1 are in red font, and antibody residues are in black font. (**A**): distance trajectory between K120/N120 and T28/K28 residues. (**B**): distance trajectory between E116 and T28/K28 residues. (**C**): distance trajectory between N185 and T28/K28. (**D**): distance trajectory between N119 and Y32residues. (**E**): distance trajectory between D117 and Y32/F32 residues. (**F**): distance trajectory between Q226 and R55 residues.

Our simulations revealed the remarkable dynamical nature of JOVI-1’s binding with TRBC1/TRBC2 for stable complexes as well. As can be seen in Figure 2, the hydrogen bonds network formed between the TRBC1 b-chain and JOVI-1 antibody changes at different simulation times. The hydrogen bonds observed in the crystal structures fluctuated or disappeared, while new hydrogen bonds that were not seen in the crystal structure formed during MD simulations. One of the most striking features is that the hydrogen bond between K120 in TRBC1 and T28 in JOVI-1 is subjected to the competition from nearby E116’s interaction with T28 (with both side chain and backbone amide NH; Figure 2 and Figure 3A,B). After stabilizing for around 400 ns, the E116-T28 becomes highly flexible; however, its interactions with T28 in mut1a and TRBC2 binding frequently form and leave (Figure 3A). N185 in TRBC forms dynamic hydrogens with T28/K28 residue in antibodies, even for the T28 with a shorter side chain and an initial 9 Å separation in the crystal structure (Figure 2B and Figure 3C).

**Figure 4:**
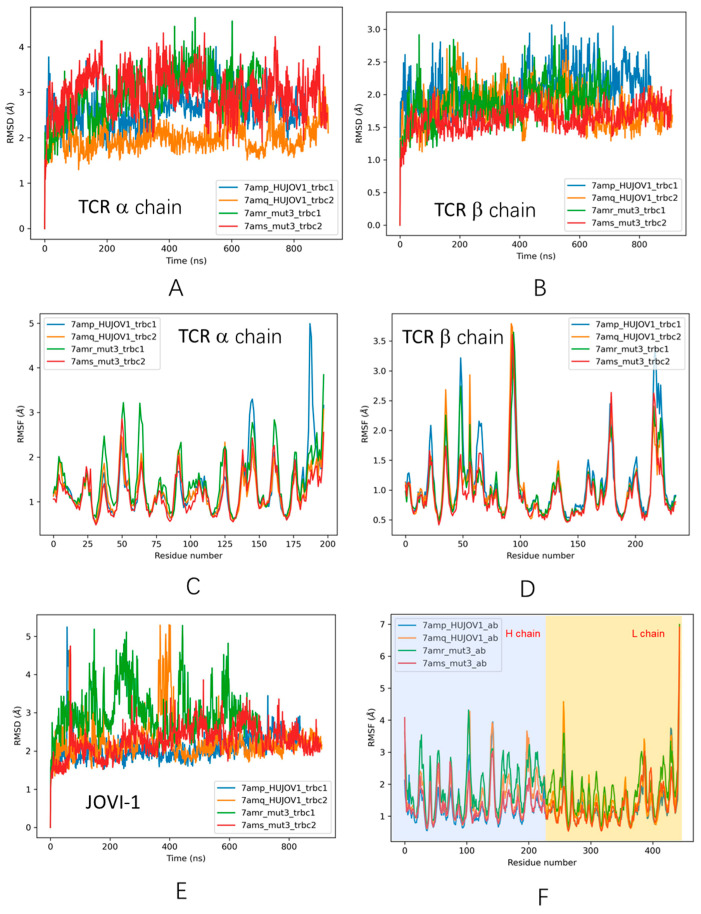
Trajectories of structural fluctuations during MD simulations. (**A**) RMSD of TCR a chain. (**B**) RMSD of TCR b chain. (**C**) RMSF of TCR a chain. (**D**) RMSF of TCR b chain; (**E**) RMSD of JOVI-1 antibodies. (**F**) RMSF of JOVI-1 antibodies.

N119 forms a hydrogen bond with Y32 in the crystal structure; the JOVI-1 can maintain the hydrogen bonding interaction with N119 frequently during simulation (Figure 2B and Figure 3D). Interestingly, this hydrogen bond is very stable in the JOVI-1/T28K TRBC1 complex (mut1a, purple line in Figure 3D). As initially observed in the crystal structure, Y32 forms a strong hydrogen bond with D117. Again, this interaction is stronger in mut1a and in the parent wt complex (Figure 4E). With the Y32F mutation in the KNF mutant, the F32 can stay near D117, mainly due to the hydrophobic part of the L119 sidechain in TRBC2.

There is one specific interaction between light chain Y37 and TRBC D227 in the JOVI-1 crystal structures. This is also a very dynamic interaction since the D227 is within the most flexible FG loop in the TCR b chain [37,38]. Not surprisingly, during MD simulations, we have observed that other FG loop residues, E223, W224, and Q226, form hydrogen bonds with Y54, R55, R59, and G62. Figure 2 highlights hydrogen bonds between Q226-R55 and E223-R59, and Figure 3F shows the trajectories of R55 binding with the backbone carbonyl group of Q226.

We compared the structural changes during simulation for four systems with known crystal structures (Figure 4). Two of the systems disassociated during simulation, corresponding to the preferred antibody–TRBC selectivity, i.e., JOVI-1 TRBC2 (7amq) and JOVI-1/KFN TRBC1 systems. As can be seen in Figure 4, there is no trend for RMSD of TCR a chain (Figure 4A), but RMSDs of TCR b chain are smaller for unstable complex (Figure 4B), indicating that the weak antibody–antigen interactions cause smaller perturbation of TRBC1/2. Examining antibodies shows the opposite trend; weak binding antibodies have larger RMSD and RMSF than stable complexes (JOVI-1 TRBC1 7amp and JOVI-1/KFN TRBC2 7ams systems). The underlying mechanism could be that the JOVI-1 antibodies have high fluctuating entropy, and the entropy can be effectively transferred to the TRBC antigen in the strong complex, while in the weak complex, antibodies maintain their high entropy.

**Figure 5:**
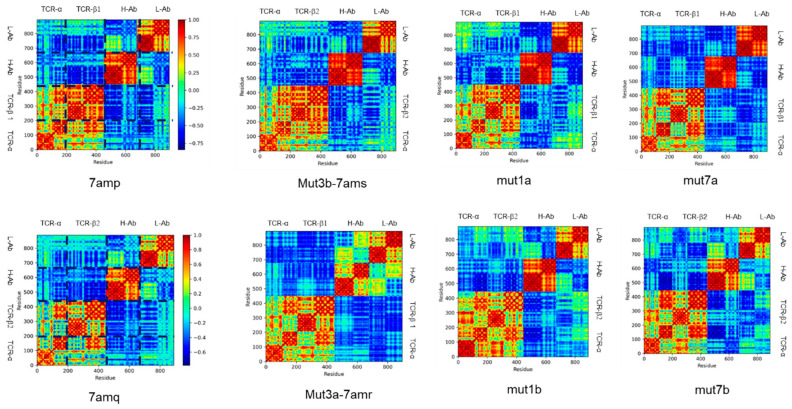
Covariance matrixes for stable complexes (**upper panels**) and unstable complexes (**lower panel**). TCR-a and TCR-b are two TCR chains. H-Ab is an antibody heavy chain, and L-Ab is an antibody light chain.

Our previous work [14] indicated that the communication between antibody and antigen could be reflected in the amino acid residue covariance matrix, which measures the motion correlations between any two residues in a protein complex. Figure 5 compares the covariance matrixes for the antibody–TRBC system with higher affinities (upper panel) and lower affinities (lower panel). While there are notable differences in correlations between antibodies and antigens, one can see that the correlations within antibodies have different features for the stronger binders and weaker ones: (1) the correlations between the constant domain and variable domain for both heavy and light chains (red squares corresponding to H-Ab/H-Ab and L-Ab/L-Ab) are more positive in the upper panel than in the lower panel; and (2) the correlations between heavy and light chain (blue and yellow squares corresponding to H-Ab/L-Ab and L-Ab/H-Ab) are more negative in the upper panel than in the lower panel.

## 4. Discussion and Conclusions

We have studied JOVI-1 family antibodies recognition of TRBC1 and TRBC2 using molecular simulations and deep learning prediction. The MD simulations have revealed the strong dynamical nature of antibody–antigen interaction in these systems.

Our Digiwiser AI model harnessed deep learning techniques for predicting affinity changes upon antibody mutations. We observed an improved correlation between the predicted score and experimental measurements when compared to traditional in silico approaches, which is attributed to the network design of the listwise ranking model.

When applied to TRBC1/2 antibody variants, we observed distinct correlation patterns on model predictions. For the TCBR1 group, this model yields a better Pearson correlation (Pearson = 0.543) compared to the weaker correlation (Pearson = 0.272) of TRBC2. Conversely, the Spearman score for the TRBC2 group demonstrates robust ranking performance (Spearman = 0.899) compared to TRBC1 (Spearman = 0.143). Due to the inherent antibody–antigen pairs of our training data, we hypothesized that such disparity between TRBC groups might be due to this model’s heightened sensitivity for differences in antibodies rather than antigens, and therefore, leads to similar scores for identical antibody mutants binding with TRBC1 and TRBC2, two identical antigens. It is also important to acknowledge that the small sample size of TRBC1/2 may also lead to the difference between groups.

A common difficulty in antibody machine-learning training is the limited availability of experimental data. It is possible to overfit a model on small training datasets with a limited diversity of protein families. From a modeling perspective, it is important to note that our approach involves constructing the model with a listwise strategy, which entails treating each group of antibodies and mutations that bind to a specific antigen as separate, independent lists during the training process. This design is particularly effective when it comes to ranking tasks involving a single antigen and multiple antibody mutations. Yet, it could also result in reduced sensitivity when dealing with antibodies that bind to different antigens, leading to a weak correlation when combining all antibodies’ affinities toward TRBC1 and TRBC2. In addition, while this approach exclusively incorporated complex sequence information and eliminated the need for structural data, we found that other structural approaches are needed to complementarily detect small variations in antigens.

Previously, Mason et al. used deep-sequenced libraries of the therapeutic antibody trastuzumab to improve antibodies’ specificity to HER2 and used more than 10,000 variants as a training set to optimize a neural network [39]. Similarly, Makowski and colleagues developed an ML model to optimize the affinity and specificity of antibodies [40], with a dataset generated in-house by sorting and deep sequencing of a mutation antibody library. This type of approach still requires a tremendous amount of experimental involvement, and more accurate and less experimentally dependent methods are needed, with possible promises for the improvement of antibody activity and properties using protein language models [41].

The free energy perturbation results reflect the effects of different types of amino acid mutations, although the errors are larger for TRBC2. It should be noted that the NAMD-FEP sampling time window here is only 1 ns, and a longer simulation time can help improve the accuracy of FEP calculation. In addition, there are several systems with charge changes in this FEP, such as T28K. For systems with charge changes, in order to maintain the electroneutrality of the system, it is necessary to change the charges of the corresponding number and sign of count ions (such as Na^+^ or Cl^−^) in FEP so that the total charge of the system in each window is zero. In order to avoid sudden changes in Coulomb interaction energy during FEP, soft-core potentials need to be used in FEP. At the same time, using smaller lambda windows near 0 and 1 can also achieve a smoother transition.

The hydrogen bond network between TRBC1 β-chain and JOVI-1 antibody changes at different simulation times, and the most obvious feature is that the hydrogen bond between K120 in TRBC1 and T28 in JOVI-1 is affected by the competition of the interaction between E116 and T28 nearby. The different hydrogen bonding network changes are presented in the simulations as differences in the dynamic binding of the individual antibodies to TRBC1 and TRBC2. Prolonged molecular dynamics simulations also observed a number of antibodies and TRBCs separating from each other from the binding site after a period of time, with poorer binding stability correlating with a poorer affinity of the antibody–antigen. The entropy effect could explain the dynamics of the different antibodies. The high fluctuation entropy of the JOVI-1 antibody has different entropy shifts in strongly and weakly bound complexes, which is ultimately reflected in a decrease in the fluctuation of the antibody itself.

In conclusion, the current model exhibits significant potential in predicting the impact of single/multiple amino acid changes, and it would be beneficial to incorporate antigens with high identity in the training set for better robustness and broader applicability. Still, our work suggests that the AI method needs to be combined with molecular simulations to computationally provide comprehensive pictures of antibody–antigen interaction, especially for systems with high dynamic interactions. For the protein–protein with large conformation dynamics contribution, more accurate free energy perturbation protocols are also needed.

## Figures and Tables

**Table 1:** Performance of several models in antibody affinity ΔΔG prediction ^1^.

Method	Pearson	Spearman
bASA	0.22	
DFIRE	0.31	
dDFIRE	0.19	
Rosetta	0.16	
STATIUM	0.32	
FoldX	0.34	
Discovery Studio	0.45	
Our Digiwiser Model	0.74–0.89	0.72–0.78

^1^ Correlations from other methods were taken from reference [28].

**Table 2:** List of simulated antibody–antigen complexes ^1^.

System	PDB Code	Antibody	Antigen	Affinity	Atoms	Stable Time ^1^	
						Close	Far
wt	7amp	HuJovi-1 Fab	TRBC1	−11.8	344,304	>850 ns	>850 ns
Wt2	7amq	HuJovi-1 Fab	TRBC2	−6.7	325,267	530 ns	550 ns
Mut1a	7amp/T28K	HuJovi-1/T28K	TRBC1	−11.2	344,248	>860 ns	>860 ns
Mut1b	7amq/T28K	HuJovi-1/T28K	TRBC2	−7.0	325,262	65 ns	250 ns
Mut2a	7amr/N96A	HuJovi-1/T28K/Y32F	TRBC1	−8.8	325,251	>900 ns	>900 ns
Mut2b	7ams/N96A	HuJovi-1/T28K/Y32F	TRBC2	−8.2	325,180	>900 ns	>900 ns
Mut3a	7amr	HuJovi-1/KFN	TRBC1	−7.8	325,394	125 ns	150 ns
Mut3b	7ams	HuJovi-1/KFN	TRBC2	−8.7	325,428	>900 ns	>900 ns
Mut4a	7amr/K28R	HuJovi-1/RFN	TRBC1	−8.0	325,606	>900 ns	>900 ns
Mut4b	7ams/K28R	HuJovi-1/RFN	TRBC2	−8.6	325,289	>900 ns	>900 ns
Mut5a	7amp/Y32F	HuJovi-1/Y32F	TRBC1	−8.7	344,366	>900 ns	>900 ns
Mut5b	7amq/Y32F	HuJovi-1/Y32F	TRBC2	-	325,344	>900 ns	>900 ns
Mut6a	7amp/A96N	HuJovi-1/A96N	TRBC1	−10.8	344,212	320 ns	>900 ns
Mut6b	7amq/A96N	HuJovi-1/A96N	TRBC2	−8.8	325,193	550 ns	600 ns
Mut7a	7amr/F32Y	HuJovi-1/T28K/A96N	TRBC1	−10.2	344,405	350 ns	>900 ns
Mut7b	7ams/F32Y	HuJovi-1/T28K/A96N	TRBC2	−8.8	325,311	30 ns	>900 ns

^1^ Close: Initial paratope–epitope amino acid pair broken, but antibody–antigen remains in contact; Far: antibody–antigen disassociated.

**Table 3:** Antibody mutation effects on antibody–antigen affinity (kcal/mol) rank.

System	Antigen	Affinity	Rank ^1^	System	Antigen	Affinity	Rank ^2^
Mut1a	TRBC1	−11.2	0.408	Mut1b	TRBC2	−7.0	0.408
Mut2a	TRBC1	−8.8	0.708	Mut2b	TRBC2	−8.2	0.713
Mut4a	TRBC1	−8.0	0.394	Mut4b	TRBC2	−8.6	0.404
Mut5a	TRBC2	−8.7	0.532				
Mut6a	TRBC1	−10.8	0.144	Mut6b	TRBC2	−8.8	0.139
Mut7a	TRBC1	−10.2	0.396	Mut7b	TRBC2	−8.8	0.398
Pearson	0.543			Pearson	0.272		
Spearman	0.143			Spearman	0.899		

^1^ Relative to HuJovi-1 Fab-TRBC1 complex; ^2^ Relative to HuJovi-1-KFN-TRBC2 complex.

**Table 4:** Free energy perturbation of antibody mutation effects (kcal/mol).

Mut	System	Antigen	ΔΔG_exp_	ΔΔG_fep_	|err|	Mut	System	Antigen	ΔΔG_exp_	ΔΔG_fep_	|err|
7AMP:T28K	Mut1a	TRBC1	0.56	0.47	0.1	7AMQ:T28K	Mut1b	TRBC2	−0.33	−2.19	1.85
7AMR(N96A)	Mut2a	TRBC1	−0.97	−3.19	2.22	7AMS:N96A	Mut2b	TRBC2	0.44	0.11	0.33
7AMR:K28R	Mut4a	TRBC1	−0.19	−0.32	0.13	7AMS:K28R	Mut4b	TRBC2	0.11	−2.47	2.58
7AMP:Y32F	Mut5a	TRBC1	3.04	2.24	0.80	7AMQ:Y32F	Mut5b	TRBC2	NB	10.27	
7AMP:A96N	Mut6a	TRBC1	0.99	0.27	0.72	7AMQ:A96N	Mut6b	TRBC2	−2.11	−0.10	2.01
7AMR:F32Y	Mut7a	TRBC1	−2.38	1.35	3.72	7AMS:F32Y	Mut7b	TRBC2	−3.78	−0.12	3.66
Average err		1.28									2.09

## Data Availability

The data used to support the findings of this study can be made available by the corresponding author upon request.
